# Occupational solar exposure and basal cell carcinoma. A review of the epidemiologic literature with meta-analysis focusing on particular methodological aspects

**DOI:** 10.1007/s10654-023-01061-w

**Published:** 2024-01-03

**Authors:** Andrea Wendt, Matthias Möhner

**Affiliations:** Federal Institute for Occupational Safety and Health, Nöldnerstr. 40-42, 10317 Berlin, Germany

**Keywords:** Basal cell carcinoma, Occupational exposure, Ultraviolet Radiation, Review, Meta-analysis

## Abstract

**Background:**

Numerous epidemiologic studies and a few systematic reviews have investigated the association between occupational solar exposure and basal cell carcinoma (BCC). However, previous reviews have several deficits with regard to included and excluded studies/risk estimates and the assessment of risk of selection bias (RoSB). Our aim was to review epidemiologic studies with a focus on these deficits and to use meta-(regression) analyses to summarize risk estimates.

**Methods:**

We systematically searched PubMed (including MEDLINE) and Embase for epidemiologic studies. Study evaluation considered four main aspects of risk of bias assessments, i.e. Selection of subjects (selection bias); Exposure variables; Outcome variables; Data analysis.

**Results:**

Of 56 identified references, 32 were used for meta-(regression) analyses. The overall pooled risk estimate for BCC comparing high/present vs. low/absent occupational solar exposure was 1.20 (95% CI 1.02–1.43); among studies without major deficits regarding data analysis, it was 1.10 (95% CI 0.91–1.33). Studies with low and high RoSB had pooled risk estimates of 0.83 (95% CI 0.73–0.93) and 1.95 (95% CI 1.42–2.67), respectively. The definitions of exposure and outcome variables were not correlated with study risk estimates. Studies with low RoSB in populations with the same latitude or lower than Germany had a pooled risk estimate of 1.01 (95% CI 0.88–1.15).

**Conclusion:**

Due to the different associations between occupational solar exposure and BCC among studies with low and high RoSB, we reason that the current epidemiologic evidence base does not permit the conclusion that regular outdoor workers have an increased risk of BCC.

**Supplementary Information:**

The online version contains supplementary material available at 10.1007/s10654-023-01061-w.

## Introduction

Natural ultraviolet (UV) light is an important risk factor for skin cancer. However, the exposure patterns that are associated with increased risks of the different skin cancer types seem to be different. Malignant melanoma is associated with intermittent exposure during recreation, particularly in childhood and adolescence. Similarly, basal cell carcinoma (BCC) seems to be foremost associated with intermittent exposure, whereas squamous cell carcinoma (SCC) is associated with total or occupational exposure [1].

Numerous epidemiologic studies have been conducted on the question whether occupational solar exposure increases BCC risk, and three systematic reviews are available [2–4]. However, only one review investigated risk estimates in dependence on risk of bias (RoB) of the underlying studies [4], focusing on BCC and SCC together. In any case, all three reviews missed important issues in their performed RoB assessments with respect to selection bias.

In occupational epidemiology, potential risk factors are usually related to blue-collar or manual workers. However, such workers that in general have a comparably low socio-economic status (SES) take part in epidemiologic studies comparably infrequent [5, 6]. In case-control studies, this concerns foremost the control group [6]. In the event of low participation rates, a biased risk estimate of the exposure-disease association can result [7]. Furthermore, selection bias can also result when the source of control participants does not represent the general population. Some studies on occupational solar exposure and BCC recruited controls from patients with non-malignant dermatologic conditions or attending skin cancer screening. However, subjects with low SES, blue-collar or outdoor jobs report non-malignant dermatologic conditions or the consultation of dermatologists and the utilization of skin cancer screening comparably infrequent [8–11].

A second concern applies to the type of exposure variables. All previous systematic reviews used some risk estimates from original studies that do not refer to usual outdoor work but to a rather intermittent type of exposure in subjects that help their relatives in farming during summer. Furthermore, they partly used risk estimates for very specific agricultural job subgroups with increased risks, while not considering the picture for agricultural jobs overall [2, 3].

Thirdly, all previous reviews missed relevant literature. For example, one review restricted studies to those that were conducted in only one country [3], while another review excluded some studies that compared specific single outdoor jobs with all other jobs/the general population [4]. For the third review, it seems that the search string used for PubMed (including MEDLINE) was rather insensitive, with only 189 hits received [2].

Our aim was to review epidemiologic studies on regular occupational solar exposure and BCC and to use meta-(regression) analyses to summarize study risk estimates. We ran a new literature search to ensure identification of relevant studies. We summarized study risk estimates depending on various aspects regarding RoB, considering selection of subjects (selection bias), exposure variables, outcome variables, and data analysis.

## Materials and methods

### Scope of the review

This review was not pre-registered. It has evolved as part of our routine work that encompassed reviewing the evidence on occupational solar exposure and BCC risk. We started by reviewing systematic reviews but noted that these had important deficits (see Introduction). Our focus was to elaborate on these deficits and to review epidemiologic studies considering these deficits.

### Literature search

We searched PubMed (including MEDLINE) in October 2021 and Embase in November 2021, adapting the search string proposed by [2] in order to increase sensitivity (Online Resource 1). In accordance with [2], studies in which the exposure was defined as work in any or a specific outdoor job or related sun/UV exposure were eligible. References were screened according to the PECOS scheme (Online Resource 2). We also inspected systematic reviews [2–4] and reference lists of original studies. Studies with full texts other than English or German were translated with DeepL (https://www.deepl.com/translator) or Google Translator (https://translate.google.com).

### Evaluation of studies

The evaluation of studies centred on four main aspects that are usually addressed in RoB assessments (e.g. [12]), i.e. (1) Selection of subjects (selection bias); (2) Exposure variables; (3) Outcome variables; (4) Data analysis.

#### Selection of subjects (selection bias)

As pointed out in the Introduction, the frequent, comparably low attendance of subjects with low SES or manual/blue-collar jobs in control groups is associated with selection bias in occupational case-control studies. In cohort studies, selection bias during follow up can occur when continued participation is a common effect of exposure and outcome [7, 13]. Moreover, selection bias can result if exposure information is missing selectively or if study groups do not stem from the same base population. In case-control studies, the latter can particularly be assumed if sources of controls do not represent the general population.

Based on available RoB assessment instruments (e.g. [12]) and the information given above, we developed a simple scheme and allocated a high RoSB when.

- in case-control studies, participation rates were unknown or < 50%^1^ in cases and/or controls
- in cohort studies, loss to follow-up was ≥ 50%.
- the availability of exposure information was < 50% among designated study participants.
- study groups did not represent the same base population; for case-control studies this was assumed for mainly dermatologic controls or other sources of controls that do presumably not represent the general population.

#### Exposure variables

WHO/ILO working group [4] excluded studies that compared specific single outdoor jobs with all other jobs/the general population. Their argument was that, in such studies, exposure reference groups also contain outdoor workers and, thus, risk estimates are underestimated. In contrast, we did not exclude such studies and aimed to evaluate whether their risk estimates differ from the estimates of other studies. We further assessed whether studies defined quantitative exposure variables, including cumulative or mean estimates of exposure, or not. Finally, these two aspects were combined, differentiating between studies with quantitative exposure variables that do not compare single outdoor jobs with all other jobs/the general population and all other studies.

#### Outcome variables

For all included studies, histological verification of the outcome can be assumed (based on pathology, medical or cancer registry records). We evaluated whether risk estimates differed between studies that involved only cases with first ever BCC and studies that potentially included cases with subsequent BCC. In the event of subsequent BCC diagnoses, risk estimates are potentially biased as the exposure period extends until after the first diagnosis. Moreover, cases possibly change their behaviour after an initial diagnosis.

#### Data analysis

As a minimum requirement, risk estimates should be controlled for age, sex, and study centre (if applicable) in a statistical (regression) model of the exposure-disease association. We also looked for other model-misspecifications, e.g. the inclusion of potentially highly correlated variables in the same regression model.

### Meta-(regression) analyses

Random-effects meta-(regression) analyses were carried out with Stata 17 [14]. Certain details are described in Online Resource 3 (e.g. reasons for excluded studies; detailed approach of selection of risk estimates). Very briefly, risk estimates for occupational solar exposure due to overall outdoor work were preferred. Otherwise, in accordance with [2], risk estimates for specific single occupations were used. However, we used only risk estimates for agricultural jobs as these were evaluated in all studies on specific occupations that were eligible for our meta-analysis and as these entail many outdoor workers. This approach ensures a certain homogeneity with regard to the index exposure among the studies on specific occupations/jobs.

### Course of analyses

After a first meta-analysis (***level 1***), studies with deficits regarding data analysis (see Material and Methods) were excluded to remove possible data analysis-related bias ahead of further analyses. At ***level 2***, several sub-analyses were conducted to investigate risk estimates with regard to: Selection of subjects (selection bias); Exposure variables; Outcome variables (see Material and Methods); Study type; Sex; Mean geographical latitude of studies (in analogy to [2]).

## Results

### Literature search

The literature search yielded 4039 hits, including 281 duplicates (Online Resource 4). Of 56 retained full texts, 32 were used for meta-(regression) analyses. The 24 excluded references are described in Online Resource 5, section A, together with one reference used in [2] that did not meet our selection criteria [15].

### Evaluation of the literature

Table 1 visualizes the evaluation results of the studies that were included in the meta-(regression) analyses. Online Resource 6 explains the results in detail. A comprehensive overview of the studies is presented in Online Resource 7 (case-control studies) and Online Resource 8 (other study types).

### Meta-(regression) analyses

**Fig. 1:**
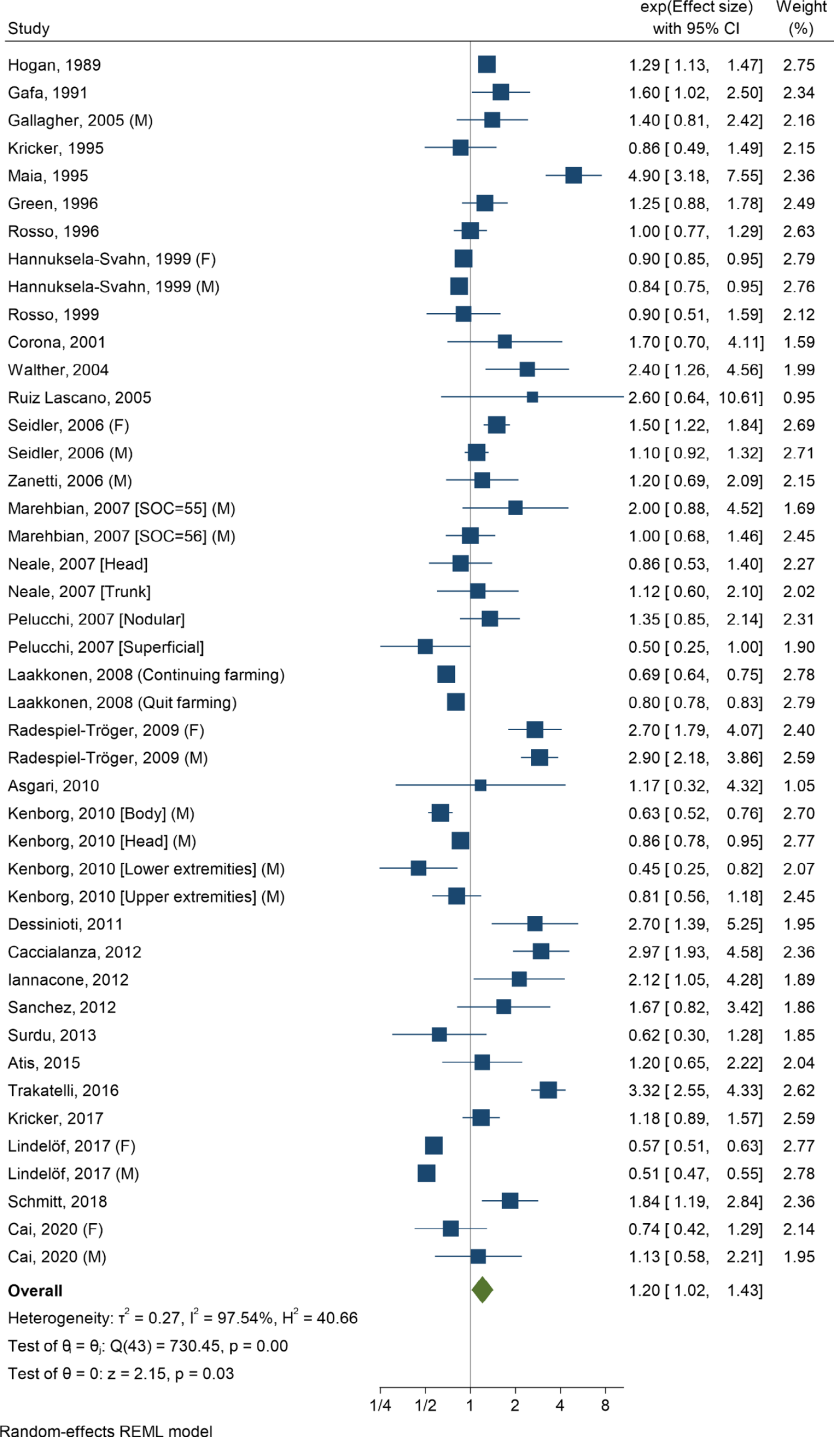
Meta-analysis of all 32 studies on the association between occupational solar exposure and the risk of basal cell carcinoma F = Females, M = Males, SOC = Standard Occupational Classification

**Fig. 2:**
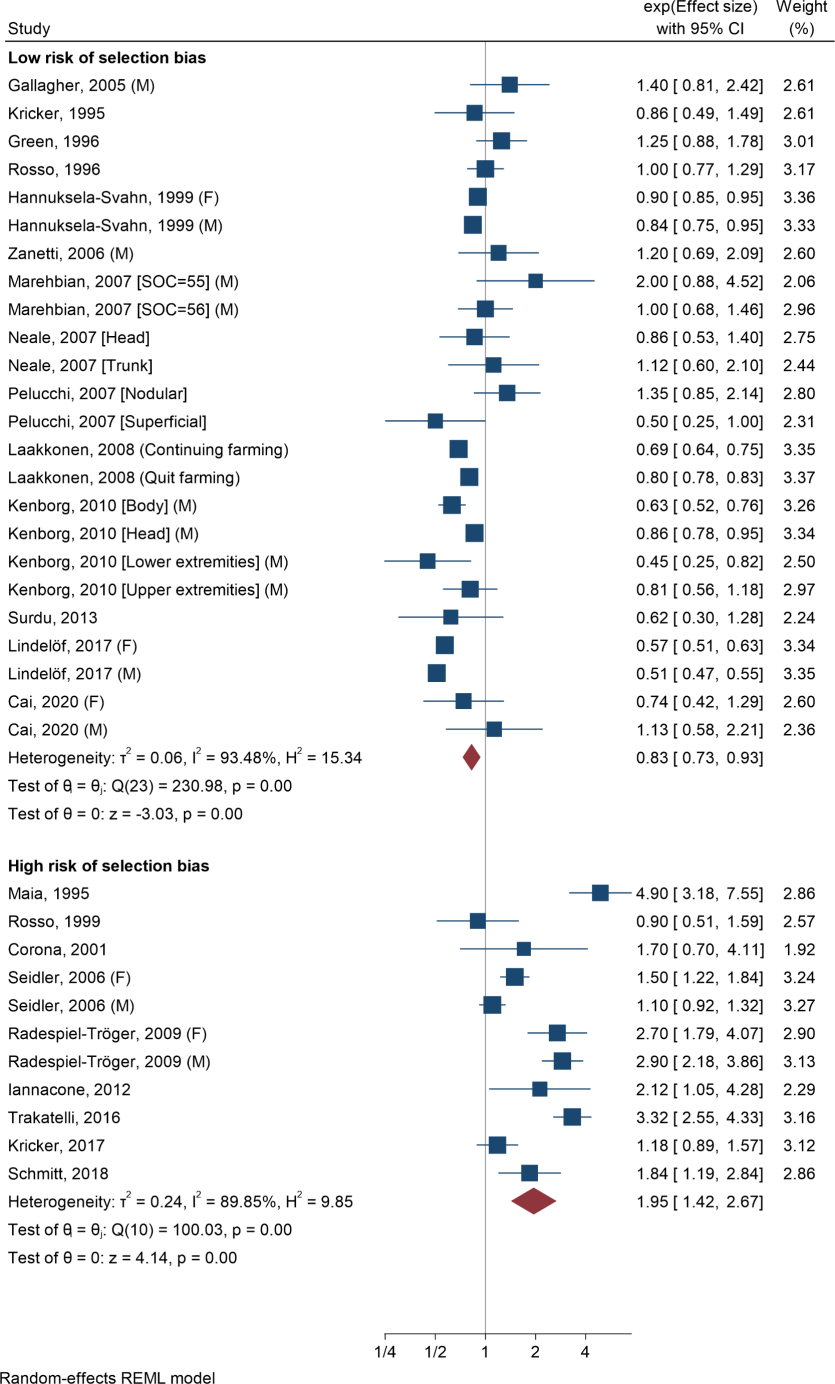
Meta-analyses of 23 studies without major deficits regarding data analysis on the association between occupational solar exposure and the risk of basal cell carcinoma, stratified by studies with low and high risk of selection bias F = Females, M = Males, SOC = Standard Occupational Classification

The first meta-analysis yielded a pooled risk estimate of 1.20 (95% CI 1.02–1.43) (level 1) (Fig. 1). At level 2, the pooled risk estimate without studies with deficits regarding data analysis was 1.10 (95% CI 0.91–1.33). For studies with low and high RoSB, the pooled risk estimates were 0.83 (95% CI 0.73–0.93) and 1.95 (95% CI 1.42–2.67), respectively (Fig. 2). Among the 16 case-control studies only, almost identical results occurred (data not shown). A stratified analysis with these studies shows that with respect to the issues used to evaluate RoSB, similar patterns emerged as for RoSB overall (Online Resource 9). The pooled risk estimate among the five cohort studies (all with low RoSB) was 0.84 (95% CI 0.75–0.95). The definitions of exposure variables and the outcome were not correlated with risk estimates (Online Resource 10). This also held true among studies with low RoSB. In this subgroup, only latitude was related to the size of risk estimates (Table 2). Studies with populations north of Germany (> 50th latitude) showed a lower pooled risk estimate (0.73; 95% CI 0.63–0.84) than studies in populations ≤ 50th latitude (1.01; 95% CI 0.88–1.15).

**Table 1:** Evaluation results for the 32 studies that were included in the meta-(regression) analyses

Study	Data analysis	Selections bias						Exposure		Outcome	Strata in meta-analysis; Analyzed comparison
		Participation rate^a^/Follow-up rate		Controls/Comparison group		Exposure complete	Overall	Exposure reference^b^	quantitative	Explicitly first BCC in cases	
**Cancer registry-based study**											
Radespiel-Tröger et al. 2009 [16] (Cohort design)	**+**			^**c**^		−	**−**	**+**	**−**	**+**	Men: longest (or, if not available, last) occupation outdoor- vs. indoor; Women; outdoor/indoor- vs. indoor
Seidler et al. 2006 [17] (Case-control design)	**+**			other cancer cases		−	**−**	**+**	**−**	**−**	Men; Women; longest/last occupation farmer/farm helper vs. “white-collar”-jobs/indoor production jobs
**Cohort study**											
Cai et al. 2020 [18]	**+**	99.89%	+	population-based	+	+	**+**	**−**	**−**	**+**	Men; Women; agricultural jobs/fishery/forestry vs. all other jobs
Laakkonen and Pukkala 2008 [19]	**+**	100%	+	population-based	+	+	**+**	**−**	**−**	**−**	Farmers from 1978 until at least 1990/1994 vs. general population; Farmers from 1978 until before 1990/1994 vs. general population
Neale et al. 2007 [20]	**+**	100%	+	population-based	+	+	**+**	**+**	**−**	**−**	BCC at head/neck; BCC at trunk; Jobs in life mainly outdoor vs. indoor
Hannuksela-Svahn et al. 1999 [21]	**+**	100%	+	population-based	+	+	**+**	**−**	**−**	**−**	Men; Women; Agriculture/Fishery/Forestry vs. general population
Green et al. 1996 [22]	**+**	80%	+	population-based	+	+	**+**	**+**	**−**	**−**	Mainly outdoor- vs. mainly indoor occupations in life
**Case-control study**											
Schmitt et al. 2018 [23]	**+** ^**d**^	21%	−	population-based	+	+	**−**	**+**	**+**	**+**	≥ 5,870.5 vs. 0 standard erythema dosage
Kricker et al. 2017 [24]	**+**	31%	−	population-based	+	+	**−**	**+**	**+**	**+**	30 + vs. 0 years outdoor work
Lindelöf et al. 2017 [25]	**+**	100%	+	population-based	+	+	**+**	**+**	**−**	**−**	Men; Women; primary occupation between 31 and 50 years of age farmer/forester/gardener vs. clerical worker
Trakatelli et al. 2016 [26]	**+**	unknown	−	dermatological; not representative	−	+	**−**	**+**	**+**	**−**	> 5 vs. 0 years outdoor work
Atis et al. 2015 [27]	**−**	unknown	−	not representative (volunteers)	−	+	**−**	**+**	**−**	**−**	outdoor work yes vs. no
Surdu et al. 2013 [28]	**+**	90%	+	hospital-based	+	+	**+**	**+**	**+**	**−**	> 5075 vs. 0 h occupational exposure
Caccialanza et al. 2012 [29]	**−**	unknown	−	dermatological	−	+	**−**	**+**	**−**	**−**	occupational exposure of at least 6 months yes vs. no
Iannacone et al. 2012 [30]	**+**	49.7%	−	dermatological (skin cancer screening)	−	+	**−**	**+**	**+**	**−**	outdoor work > 10 vs. 0 years
Sánchez et al. 2012 [31]	**−**	unknown	−	dermatological	−	+	**−**	**+**	**−**	**−**	occupational outdoor activity at age > 30 years yes vs. no
Dessinioti et al. 2011 [32]	**−**	unknown	−	not representative; hospital-based; dermatological	−	+	**−**	**+**	**+**	**−**	> 5 vs. 0–5 years outdoor-work
Asgari et al. 2010 [33]	**−**	100%	+	population-based	+	+	**+**	**+**	**−**	**−**	Occupational sun exposure high vs. low (based on occupations)
Kenborg et al. 2010 [34]	**+**	100%	+	population-based	+	+	**+**	**+**	**+**	**+**	Men; BCC at head; trunk; upper extremities; lower extremities; >10 vs. <1 years outdoor work
Marehbian et al. 2007 [35]	**+**	68%	+	population-based	+	+	**+**	**−**	**−**	**−**	Men; ever vs. never Farm owner/manager; other agricultural occupations
Pelucchi et al. 2007 [36]	**+**	97%	+	hospital-based	+	+	**+**	**+**	**+**	**+**	nodular BCC; superficial BCC; >median vs. 0 h occupational exposure weighed for clothes
Zanetti et al. 2006 [37]	**+**	92.8%	+	hospital-based	+	+	**+**	**+**	**+**	**+**	Men; 3878 + hours vs. never outdoor work
Ruiz Lascano et al. 2005 [38]	**−**	99%	+	hospital-based	+	+	**+**	**+**	**−**	**−**	Occupational sun exposure high/moderate vs. low
Walther et al. 2004 [39]	**−**	unknown	−	hospital-based (incl. some dermatological)	+	+	**−**	**+**	**−**	**−**	frequent/sometimes vs. seldom/no occupational exposure
Corona et al. 2001 [40]	**+**	unknown	−	dermatological	−	+	**−**	**+**	**+**	**−**	> 8 vs. ≤8 years outdoor work
Rosso et al. 1999 [41]	**+**	81%	+	not representative	−	+	**−**	**+**	**+**	**−**	77 200 + vs. 0 h outdoor work
Rosso et al. 1996 [42]	**+**	73.6%	+	population-based; hospital-based	+	+	**+**	**+**	**+**	**−**	54 720 + vs. <7200 h outdoor work
Gallagher et al. 1995 [43]	**+**	71%	+	population-based	+	+	**+**	**+**	**+**	**−**	Men; ≥105 vs. <15 h/year occupational exposure weighed for clothes worn
Maia et al. 1995 [44]	**+**	unknown	−	dermatological	−	+	**−**	**−**	**−**	**−**	ever vs. never activity in agriculture
Kricker et al. 1995 [45]	**+**	89%	+	population-based	+	+	**+**	**+**	**+**	**−**	≥ 49.4 vs. ≤14.7 h/week occupational exposure
Gafà et al. 1991 [46]	**−**	unknown	−	not representative; hospital-based (possibly dermatological)	−	+	**−**	**−**	**+**	**−**	≥ 10 vs. <10 years work in agriculture
Hogan et al. 1989 [47]	**−**	43.7%	−	population-based	+	+	**−**	**−**	**−**	**−**	Occupation as farmer yes vs. no

**+** Study fulfills criterion (for definition, see Material and Methods); **−** Study does not fulfill criterion

^**a**^ The indicated participation rate for case-control studies applies to control participants

^**b**^ „–“ when a specific single outdoor occupation was compared with all other occupations or the general population; „+“ otherwise

^**c**^ Study is based on selected regions in Bavaria, Germany. No individual data for the base population available. For the analysis, the study authors weighed the population count in single years with the share of outdoor and indoor jobs in the Bavarian population and the share of available job notifications in cancer registry for registered cancer cases

^**d**^ The study reported by Schmitt et al. 2018 is multi-centric. In this publication, risk estimates were not adjusted for study-centre. Later on, several sensitivity analyses, amongst them an analysis adjusted for study centre, were conducted ([48]; Bauer A, personal communication). Thus, we classified the study as appropriate with regard to data analysis

**Table 2:** The influence of specific issues on risk estimates of studies on the association between occupational solar exposure and BCC. Results based on separate univariate meta-regression analyses, restricted to studies without major deficits regarding data analysis and with low risk of selection bias

Comparison	Risk estimate	Lower 95%-CI	Upper 95%-CI	P>|z|
Quantitative vs. other exposure variables^**a**^	1.03^**b**^	0.80	1.33	
Studies that compare specific single outdoor occupations with all other occupations/the general population vs. all other studies^**a**^	1.09	0.84	1.42	
Studies with BCC cases that were explicitly first ever BCC cases versus other studies^**a**^	0.96	0.73	1.26	
Case-control vs. cohort studies^**a**^	0.91	0.70	1.17	
Hospital- vs. population-based case-control studies^**a,c**^	1.18	0.72	1.93	
Women vs. men^**a,d**^	0.87	0.57	1.32	
Increase of latitude by one degree^**e**^	0.99	0.98	1.00	0.02

^**a**^ The categorization of studies is shown in Table 1

^**b**^ The regression coefficient and 95% confidence interval is the same for studies both with cumulative exposure variables and without exposure reference groups containing “all other occupations”/the general population as compared to all other studies

^**c**^ Study [42] was excluded from this analysis as the control group included both hospital- and population-based controls

^**d**^ This analysis used the sex-specific risk estimates from the studies [18, 21, 25, 34, 35, 37] and [43]

^**e**^ The following latitudes were allocated to the studies: 27 [20, 22], 34 [45], 36 [18], 43 [35, 36], 46 [42], 47 [28], 55 [34, 43], 59 [25] and 63 [19, 21]. The study by Zanetti et al. 2006 [37] involved subjects from Italy, Spain, France, Portugal, Denmark, Germany and Argentina. Due to the wide range of latitudes, this study was excluded from this analysis

## Discussion

### Selection of subjects (selection bias)

Our analyses indicate that studies with high RoSB overestimate underlying risk. Most of them are case-control studies with unknown or low participation rates, particularly among controls. Unfortunately, study reports do usually not contain information on the representativeness of the control group. Unless this is implemented, low or unknown participation rates should be treated with caution. The same is true for presumably unrepresentative sources of controls such as patients with minor dermatologic conditions. Most of the studies with high RoSB had more than one limitation that led to this characterization. Of course, if both a low participation and an ill-defined control group are present at the same time, it can hardly be evaluated whether one or the other or both lead to biased risk estimates.

### Diagnostic/detection bias

BCC is a condition with a certain diagnostic bias. Data from a nationwide dermatopathology laboratory in Germany show the highest mean tumor depths in members of agricultural health and local public health insurances [49]. The latter involve more people that work in physically strenuous jobs and less people that work in offices compared to the general population [50]. These findings are in accordance with observations that a lower SES or outdoor work are inversely associated with the usage of skin cancer screening [10, 11] and initial dermatologist visits [9, 10]. Such a diagnostic bias putatively is also present in the reviewed epidemiologic studies. However, as it concerns almost all studies, its impact cannot be evaluated. Yet, an underestimation of risk might be limited. As BCC is a tumor that continuously infiltrates adjacent tissue, the diagnostic bias probably concerns mainly the time point a person seeks medical help but not if someone seeks medical help or not. The tendency for delayed diagnoses in outdoor workers might even lead to an overestimation of outdoor exposure and risk. Studies including medical examination and histological verification to record all BCC cases in populations, accounting for previously diagnosed BCC and tumor depth, would help to evaluate BCC risk without this bias. Among the studies in our review, one used medical surveys to identify BCC cases. It did not show a clearly increased risk in relation to outdoor work as compared to indoor work (RR = 1.25; 95% CI 0.88–1.78) [22]. We are not aware of further published studies on occupational solar exposure and BCC that actively used medical examinations to identify cases, including surveys. In case-control studies, the medical examination to identify unknown BCC cases in the control group and the knowledge of their occupational solar exposure would also help to quantify diagnostic bias.

### Exposure variables

Due to a possible underestimation of the exposure-disease association, WHO/ILO working group [4] excluded studies that compared specific single outdoor jobs with all other jobs/the general population. However, this does not seem justified. First, it impedes the evaluation of the impact of such studies on pooled risk estimates. Our analyses did not identify an impact. Secondly, exposure reference groups are seldom free of any exposure. For example, when quantitative exposure variables are categorized, the reference levels often include certain fractions of outdoor work as well.

With regard to studies on single outdoor occupations, we focused on agricultural jobs. This might have introduced some bias. However, in addition to our primary analyses on exposure variables, a secondary stratified analysis of studies on agricultural jobs and of studies on outdoor jobs in general did not show systematic differences of risk estimates either (data not shown).

### Types of UV exposure and BCC

Our meta-analysis of studies with low RoSB yielded a summary risk estimate of 0.83 (95% CI 0.73–0.93). We would by no means interpret this inverse association as causal in the sense that the largest outdoor work exposure leads to the lowest risk of BCC. For example, personal protection measures and protective work conditions might have affected the result. However, regular outdoor workers also establish a continuous natural UV protection throughout the year at sun-exposed parts of the body and are, thus, putatively less sensitive to periods of intensive UV exposure than indoor workers. In fact, epidemiologic studies generally show that intermittent UV exposure is important for BCC occurrence [1]. Furthermore, it was shown that BCC incidence is highest in subjects with high SES [19, 25, 51–53] who work comparatively seldom in outdoor jobs.

Our review focused on regular outdoor occupations. We excluded studies that evaluated BCC risk of subjects with more intermittent and intense outdoor UV exposure. For instance, Vlajinac et al. [54] reported an increased risk of BCC in subjects that help their relatives in agriculture in summer, while regular farmers had no increased risk based on their Table 1. Additional studies should evaluate BCC risk in association with occupational exposure scenarios that encompass intermittent and intense outdoor UV exposure, such as in seasonal workers.

### Data analysis

We did not evaluate risk estimates in dependence on adjustment for potential confounders such as SES, skin type or recreational UV exposure. In fact, whether such variables are confounders is study-specific and cannot be assumed in general. Unfortunately, study reports generally lack information to judge this. Future studies should take the aspect of confounding more into account. However, for studies with high RoSB, adjustment for SES is advisable to reduce selection bias. One of the studies with high RoSB adjusted for education, but probably only in dichotomous form [30]. A separate sensitivity analysis [48] of another study with high RoSB [23] yielded a 20% lower risk estimate with versus without adjustment for education.

### Latitude of the study population

Our analyses showed a lower pooled risk estimate for studies with latitudes > 50° than for studies with latitudes ≤ 50°. This could have several reasons. In this review, studies in populations more in the north than Germany were exclusively based on exhaustive registries. This prevents potential bias that can occur in studies that actively recruit and interrogate participants. A second possible reason could be the weaker solar radiation more in the north as compared to other regions. However, the weaker radiation is accompanied by a generally lighter and more sensitive skin of the common, long-time resident population and, thus, might not be the main explanation.

### Results of previous reviews as compared to our review

Previous meta-analyses resulted in pooled risk estimates of 1.43 (95% CI 1.23–1.66) [2] and 1.50 (95% CI 1.10–2.04) [4]. In comparison, our pooled estimate based on all studies was only 1.20 (95% CI 1.02–1.43). The main reasons for this difference were already mentioned in the Introduction.

Of the previous systematic reviews, only the review by WHO/ILO working group [4] evaluated study risk estimates in dependence on RoB. The analysis for non-melanoma skin cancer (NMSC, i.e. BCC and/or SCC) showed a lower pooled risk estimate for studies with only low RoB (1.11; 95% CI 0.86–1.43) as compared to studies with a high RoB in at least one of nine domains (1.98; 95% CI 1.44–2.72) (Fig. A7.8, page 180); most of the studies with low RoB also had a low RoSB according to our criteria. A stratification of the studies from WHO/ILO working group’s meta-analysis on BCC (Fig. A6.4, page 170) would result in a pooled risk estimate of 0.98 (95% CI 0.76–1.26) for studies with low RoB (acc. to Fig. 5, page 56) and, in addition, low RoSB according to our criteria [37, 42, 43, 45] (Online Resource 11). The studies with high RoB in any of the nine domains (acc. to Fig. 5, page 56) would yield a pooled risk estimate of 1.67 (95% CI 1.12–2.49). Despite the divergent results of their stratified analysis on NMSC risk, WHO/ILO working group [4] concluded a moderate quality of evidence *for* a positive association between occupational solar exposure and NMSC. In our view, this conclusion is not justified.

### Further aspects

We did not formally investigate BCC risk related to occupational solar exposure separately for BCC at different anatomic locations, for different histologic BCC subtypes or for subjects with different skin sensitivity (skin type or tanning ability). Only few studies provided information on these questions, specifically few studies with adjustment for age and sex and with low RoSB [20, 28, 34, 36, 43, 45]. One study showed increased risks of BCC at the head/neck and of nodular BCC, especially in relation to shorter occupational solar exposure, but not of BCC at the trunk and of superficial BCC [20]. On the contrary, another study showed a positive association between occupational solar exposure and BCC at the trunk but not BCC at other locations [45]. Future studies should investigate these issues further.

Protection measures and work conditions might affect BCC risk in outdoor workers, e.g. clothes, headgear, sunscreen, sunglasses, working in shade/shading of workplaces, work breaks when UV index is highest, etc. We did not evaluate the influence of such measures on risk estimates. Indeed, this was usually not focused in the included original studies. In some studies, the exposure variables were weighted or the analyses were adjusted for some protection measures, usually clothes worn. Yet, no study stratified the analysis by protection measures/work conditions or conducted analyses using variables such as “work in intense sun” or “work in sun without protection”. Thus, present study results must be interpreted against the background of habitual work conditions and protection measures in the study populations.

## Conclusion

We reason that the current epidemiologic evidence base does not permit the conclusion that regular outdoor workers have an increased risk of BCC. Studies with low risk of bias, particularly with low risk of selection bias, do not show a positive association between occupational solar exposure and BCC. Many of the available studies on natural UV radiation and BCC rather suggest that intensive UV exposure periods during spare time and sunburns (frequently defined as “intermittent” exposure) increase risk. Future studies should investigate if the frequent observation of a higher BCC risk in subjects with a comparably high SES can be explained by intermittent intense UV radiation exposure periods that lead to erythema and sunburns. Additionally, the influence of diagnostic/detection bias on risk estimates should be quantified.

### Electronic supplementary material

Below is the link to the electronic supplementary material.

Supplementary Material 1

Supplementary Material 2

Supplementary Material 3

Supplementary Material 4

Supplementary Material 5

Supplementary Material 6

Supplementary Material 7

Supplementary Material 8

Supplementary Material 9

Supplementary Material 10

Supplementary Material 11

## Footnotes

1 There is no consensus on definitive critical proportions. Furthermore, a certain proportion of missing observations does not indicate selection bias, just as a certain proportion of available observations does not indicate the absence of selection bias. The differentiation of studies will be used in meta-(regression) analysis in order to assess whether systematic selection bias might be present or not.

## References

[1] Armstrong BK, Kricker A (2001). The epidemiology of UV induced Skin cancer. J Photochem Photobiol B.

[2] Bauer A, Diepgen TL, Schmitt J (2011). Is occupational solar ultraviolet irradiation a relevant risk factor for basal cell carcinoma? A systematic review and meta-analysis of the epidemiological literature. Br J Dermatol.

[3] Loney T, Paulo MS, Modenese A (2020). Global evidence on occupational sun exposure and keratinocyte cancers: a systematic review. Br J Dermatol.

[4] WHO/ILO working group (2021). The effect of occupational exposure to solar ultraviolet radiation on malignant skin Melanoma and nonmelanoma Skin cancer: a systematic review and meta-analysis from the WHO/ILO Joint Estimates of the work-related Burden of Disease and Injury.

[5] Rönmark E, Lundqvist A, Lundbäck B, Nyström L (1999). Non-responders to a postal questionnaire on respiratory symptoms and Diseases. Eur J Epidemiol.

[6] Möhner M (2012). The impact of selection bias due to increasing response rates among population controls in occupational case-control studies. Am J Respir Crit Care Med.

[7] Hernán MA, Hernandez-Diaz S, Robins JM (2004). A structural approach to selection bias. Epidemiology.

[8] Ofenloch RF, Schuttelaar ML, Svensson A (2019). Socioeconomic status and the prevalence of skin and atopic Diseases in five European countries. Acta Derm Venereol.

[9] Tripathi R, Knusel KD, Ezaldein HH, Scott JF, Bordeaux JS (2018). Association of Demographic and Socioeconomic Characteristics with Differences in Use of Outpatient Dermatology Services in the United States. JAMA Dermatol.

[10] Zink A, Tizek L, Schielein M, Böhner A, Biedermann T, Wildner M (2018). Different outdoor professions have different risks - a cross-sectional study comparing non-melanoma Skin cancer risk among farmers, gardeners and mountain guides. J Eur Acad Dermatol Venereol.

[11] LeBlanc WG, Vidal L, Kirsner RS (2008). Reported Skin cancer screening of US adult workers. J Am Acad Dermatol.

[12] National Toxicology Program: OHAT Risk of Bias Tool for Animal and Human Studies. National Toxicology Program, U.S. Dept. of Health and Human Services., 2015 (https://ntp.niehs.nih.gov/ntp/ohat/pubs/riskofbiastool_508.pdf).

[13] Kristman V, Manno M, Cote P (2004). Loss to follow-up in cohort studies: how much is too much?. Eur J Epidemiol.

[14] StataCorp.: Stata Statistical Software: Release 17.College Station, TX: StataCorp LLC, 2021.

[15] Tobia L, Fanelli C, Bianchi S (2007). [Professional exposure to natural ultraviolet radiation: risk assessment and management and preventing strategies]. G Ital Med Lav Ergon.

[16] Radespiel-Tröger M, Meyer M, Pfahlberg A, Lausen B, Uter W, Gefeller O (2009). Outdoor work and Skin cancer incidence: a registry-based study in Bavaria. Int Arch Occup Environ Health.

[17] Seidler A (2006). UV-exponierte Berufe Und Hauttumoren: Berufsbezogene Auswertung Von Daten Des Krebsregisters Rheinland-Pfalz. Zbl Arbeitsmed.

[18] Cai H, Sobue T, Kitamura T (2020). Epidemiology of nonmelanoma Skin cancer in Japan: occupational type, lifestyle, and family history of cancer. Cancer Sci.

[19] Laakkonen A, Pukkala E (2008). Cancer incidence among Finnish farmers, 1995–2005. Scand J Work Environ Health.

[20] Neale RE, Davis M, Pandeya N, Whiteman DC, Green AC (2007). Basal cell carcinoma on the trunk is associated with excessive sun exposure. J Am Acad Dermatol.

[21] Hannuksela-Svahn A, Pukkala E, Karvonen J (1999). Basal cell skin carcinoma and other nonmelanoma skin cancers in Finland from 1956 through 1995. Arch Dermatol.

[22] Green A, Battistutta D, Hart V, Leslie D, Weedon D (1996). Skin cancer in a subtropical Australian population: incidence and lack of association with occupation. The Nambour Study Group. Am J Epidemiol.

[23] Schmitt J, Haufe E, Trautmann F (2018). Occupational UV-Exposure is a major risk factor for basal cell carcinoma: results of the Population-based case-control study FB-181. J Occup Environ Med.

[24] Kricker A, Weber M, Sitas F (2017). Early life UV and risk of basal and Squamous Cell Carcinoma in New South Wales, Australia. Photochem Photobiol.

[25] Lindelöf B, Lapins J, Dal H (2017). Shift in Occupational Risk for basal cell carcinoma from Outdoor to indoor workers: a large Population-based case-control Register Study from Sweden. Acta Derm Venereol.

[26] Trakatelli M, Barkitzi K, Apap C, Majewski S, De Vries E (2016). Skin cancer risk in outdoor workers: a European multicenter case-control study. J Eur Acad Dermatol Venereol.

[27] Atis G, Altunay IK, Demirci GT, Aydin E, Mammadov D, Karsidag S (2015). The most common skin cancers and the risk factors in geriatric patients: a hospital based-controlled study. J Experimental Clin Med (Turkey).

[28] Surdu S, Fitzgerald EF, Bloom MS (2013). Occupational exposure to ultraviolet radiation and risk of non-melanoma Skin cancer in a multinational European study. PLoS ONE.

[29] Caccialanza M, Piccinno R, Veraldi S, Gnecchi L, Forti S (2012). Sun exposure and development of basal cell carcinomas: comparison between 504 patients affected by basal cell carcinoma and 475 non-affected. G Ital Dermatol Venereol.

[30] Iannacone MR, Wang W, Stockwell HG (2012). Patterns and timing of sunlight exposure and risk of basal cell and squamous cell carcinomas of the skin–a case-control study. BMC Cancer.

[31] Sánchez G, Nova J, de la Hoz F (2012). [Risk factors for basal cell carcinoma: a study from the national dermatology center of Colombia]. Actas Dermosifiliogr.

[32] Dessinioti C, Tzannis K, Sypsa V (2011). Epidemiologic risk factors of basal cell carcinoma development and age at onset in a southern European population from Greece. Exp Dermatol.

[33] Asgari MM, Tang J, Warton ME (2010). Association of Prediagnostic Serum Vitamin D Levels with the development of basal cell carcinoma. J Invest Dermatology.

[34] Kenborg L, Jørgensen AD, Budtz-Jørgensen E, Knudsen LE, Hansen J (2010). Occupational exposure to the sun and risk of skin and lip cancer among male wage earners in Denmark: a population-based case-control study. Cancer Causes Control.

[35] Marehbian J, Colt JS, Baris D (2007). Occupation and keratinocyte cancer risk: a population-based case-control study. Cancer Causes Control.

[36] Pelucchi C, Di Landro A, Naldi L, La Vecchia C, Oncology Study Group of the Italian Group for Epidemiologic Research in D (2007). Risk factors for histological types and anatomic sites of cutaneous basal-cell carcinoma: an Italian case-control study. J Invest Dermatol.

[37] Zanetti R, Rosso S, Martinez C (2006). Comparison of risk patterns in carcinoma and Melanoma of the skin in men: a multi-centre case-case-control study. Br J Cancer.

[38] Ruiz Lascano A, Kuznitzky R, Garay I, Ducasse C, Albertini R (2005). [Risk factors for basal cell carcinoma. Case-control study in Cordoba]. Medicina.

[39] Walther U, Kron M, Sander S (2004). Risk and protective factors for sporadic basal cell carcinoma: results of a two-centre case-control study in southern Germany. Clinical actinic elastosis may be a protective factor. Br J Dermatol.

[40] Corona R, Dogliotti E, D’Errico M (2001). Risk factors for basal cell carcinoma in a Mediterranean population: role of recreational sun exposure early in life. Arch Dermatol.

[41] Rosso S, Joris F, Zanetti R (1999). Risk of basal and squamous cell carcinomas of the skin in Sion, Switzerland: a case-control study. Tumori.

[42] Rosso S, Zanetti R, Martinez C (1996). The multicentre south European study ‘Helios’. II: different sun exposure patterns in the aetiology of basal cell and squamous cell carcinomas of the skin. Br J Cancer.

[43] Gallagher RP, Hill GB, Bajdik CD (1995). Sunlight exposure, pigmentary factors, and risk of nonmelanocytic Skin cancer. I. basal cell carcinoma. Arch Dermatol.

[44] Maia M, Proenca NG, de Moraes JC (1995). Risk factors for basal cell carcinoma: a case-control study. Rev Saude Publica.

[45] Kricker A, Armstrong BK, English DR, Heenan PJ (1995). A dose-response curve for sun exposure and basal cell carcinoma. Int J Cancer.

[46] Gafà L, Filippazzo MG, Tumino R, Dardanoni G, Lanzarone F, Dardanoni L (1991). Risk factors of nonmelanoma Skin cancer in Ragusa, Sicily: a case-control study. Cancer Causes Control.

[47] Hogan DJ, To T, Gran L, Wong D, Lane PR (1989). Risk factors for basal cell carcinoma. Int J Dermatol.

[48] Bauer A, Haufe E, Heinrich L, Seidler A, Schmitt J (2021). [Update on occupational Skin cancer—basal cell carcinoma and solar UV exposure]. Hautarzt.

[49] Schäfer I, Reusch M, Siebert J, Spehr C, Augustin M (2014). Health care characteristics of basal cell carcinoma in Germany: the role of insurance status and socio-demographic factors. J Dtsch Dermatol Ges.

[50] Epping J, Geyer S, Eberhard S, Tetzlaff J (2021). [Completely different or quite similar? The Sociodemographic structure of the AOK Lower Saxony in comparison to the General and Working Population in Lower Saxony and the Federal Republic of Germany]. Gesundheitswesen.

[51] Steding-Jessen M, Birch-Johansen F, Jensen A, Schüz J, Kjær SK, Dalton SO (2010). Socioeconomic status and non-melanoma Skin cancer: a nationwide cohort study of incidence and survival in Denmark. Cancer Epidemiol.

[52] Augustin J, Kis A, Sorbe C, Schäfer I, Augustin M (2018). Epidemiology of Skin cancer in the German population: impact of socioeconomic and geographic factors. J Eur Acad Dermatol Venereol.

[53] Corazza M, Ferretti S, Scuderi V, Borghi A. Socio-economic status and Skin cancer incidence: a population-based, cohort study in the province of Ferrara, northern Italy. Clin Exp Dermatol 2021. 10.1111/ced.14599 33577111

[54] Vlajinac HD, Adanja BJ, Lazar ZF (2000). Risk factors for basal cell carcinoma. Acta Oncol.

[55] Bauer A, Haufe E, Heinrich L (2020). Basal cell carcinoma risk and solar UV exposure in occupationally relevant anatomic sites: do histological subtype, Tumor localization and Fitzpatrick phototype play a role? A population-based case-control study. J Occup Med Toxicol.

[56] Bogavac A, Vlajinac H, Bjekic M, Adanja B, Marinkovic J, Medenica L (1998). Risk factors for basal cell carcinoma: case-control study. Arch Oncol.

[57] de Vries E, Trakatelli M, Kalabalikis D (2012). Known and potential new risk factors for Skin cancer in European populations: a multicentre case-control study. Br J Dermatol.

[58] Green A, Battistutta D (1990). Incidence and determinants of Skin cancer in a high-risk Australian population. Int J Cancer.

[59] Kaskel P, Lange U, Sander S (2015). Ultraviolet exposure and risk of Melanoma and basal cell carcinoma in Ulm and Dresden, Germany. J Eur Acad Dermatol Venereol.

[60] Lock-Andersen J, Drzewiecki KT, Wulf HC (1998). The measurement of constitutive and facultative skin pigmentation and estimation of sun exposure in caucasians with basal cell carcinoma and cutaneous malignant Melanoma. Br J Dermatol.

[61] Maksimović N, Raznatović M, Marinković J, Janković J (2006). [Exposure to sun radiation as a risk factor for the occurrence of basal cell carcinoma in the montenegrian population]. Vojnosanitetski pregled. Military-medical and Pharmaceutical Review.

[62] Janković S, Maksimović N, Janković J, Ražnatović M, Marinković J, Tomić-Spirić V (2010). Risk factors for basal cell carcinoma: results from the case-control study. Open Med.

[63] Milán T, Verkasalo PK, Kaprio J, Koskenvuo M (2003). Lifestyle differences in twin pairs discordant for basal cell carcinoma of the skin. Br J Dermatol.

[64] Naldi L, DiLandro A, D’Avanzo B, Parazzini F (2000). Host-related and environmental risk factors for cutaneous basal cell carcinoma: evidence from an Italian case-control study. J Am Acad Dermatol.

[65] Suarez B, Lopez-Abente G, Martinez C (2007). Occupation and Skin cancer: the results of the HELIOS-I multicenter case-control study. BMC Public Health.

[66] Rosso S, Zanetti R, Pippione M, Sancho-Garnier H (1998). Parallel risk assessment of Melanoma and basal cell carcinoma: skin characteristics and sun exposure. Melanoma Res.

[67] Paavilainen V, Tuominen J, Pukkala E, Saari KM (2005). Basal cell carcinoma of the eyelid in Finland during 1953-97. Acta Ophthalmol Scand.

[68] Matas-Nadal C, Sagristà M, Gómez-Arbonés X (2021). Risk factors for early-onset basal cell carcinomas and the trend towards their female predominance. JDDG - Journal of the German Society of Dermatology.

[69] Nemer KM, Bauman TM, Boyd AS (2018). Risk factors for basal cell carcinoma in men younger than 40 years: a case-control study. Dermatol Surg.

[70] Bakos RM, Kriz M, Mühlstädt M, Kunte C, Ruzicka T, Berking C (2011). Risk factors for early-onset basal cell carcinoma in a German institution. Eur J Dermatol.

[71] Marks R, Jolley D, Dorevitch AP, Selwood TS (1989). The incidence of non-melanocytic skin cancers in an Australian population: results of a five-year prospective study. Med J Aust.

[72] Gon A, Minelli L (2011). Risk factors for basal cell carcinoma in a southern Brazilian population: a case-control study. Int J Dermatol.

[73] Vornicescu C, Ungureanu L, Senila SC (2020). Assessment of sun-related behavior and serum vitamin D in basal cell carcinoma: preliminary results. Exp Ther Med.

[74] Lear JT, Tan BB, Smith AG (1997). Risk factors for basal cell carcinoma in the UK: case-control study in 806 patients. J R Soc Med.

[75] Lichte V, Dennenmoser B, Dietz K (2010). Professional risk for Skin cancer development in male mountain guides–a cross-sectional study. J Eur Acad Dermatol Venereol.

[76] Percivalle S, Piccinno R, Baratto S, Raimondi S, Caccialanza M (2005). Sun exposure and development of basal-cell carcinomas. A retrospective study of 505 patients. Skin Cancer.

[77] Rollison DE, Iannacone MR, Messina JL (2012). Case-control study of Smoking and non-melanoma Skin cancer. Cancer Causes Control.

