# Supplementary material for: Occupational solar exposure and basal cell carcinoma. A review of the epidemiologic literature with meta-analysis focusing on particular methodological aspects
Source: Eur J Epidemiol. 2024 Jan 3;39(1):13–25. doi: 10.1007/s10654-023-01061-w (PMC10810945; doi:10.1007/s10654-023-01061-w)
Supplement: Supplementary file 1 — Supplementary Material 1 [file 10654_2023_1061_MOESM1_ESM.docx]

# Online Resource 1: PubMed and Embase searches

**PubMed-Search on October 15, 2021; 2619 hits**

https://pubmed.ncbi.nlm.nih.gov/

Adapted from search strategy by Bauer et al. [2]; Combination of their substrings for “UV radiation exposure” and “work-related exposure” into one substring (#3) to conduct a search as sensitive as possible. Use of MeSH-headings where possible

1. Study type:
   epidemiological study[mh] OR incidence[mh] OR risk[mh]
2. Outcome:
   carcinoma, basal cell[mh] OR skin cancer[mh] OR skin neoplasm[mh]
3. UV radiation exposure/work-related exposure:
   ultraviolet rays[mh] OR ultraviolet light[mh] OR sunlight[mh] OR occupational OR occupation[mh] OR outdoor work OR workplace[mh] OR work[mh]
4. #1 AND #2 AND #3

**Embase-Search on November 23, 2021; 1419 hits**

https://www.embase.com/search/quick

Adapted from PubMed search strategy by translation of the MeSH terms into Emtree terms, where possible. We searched for Emtree terms that best match the concepts described by PubMed’s MeSH terms based on the studies reviewed by [2].

1. Study type:
   ‘cancer risk’/de OR 'incidence'/de OR 'risk'/de OR 'cohort analysis'/de OR ‘controlled study’/de OR 'case control study'/exp
2. Outcome:
   'basal cell carcinoma'/de OR 'non melanoma skin cancer'/de
3. UV radiation exposure/work-related exposure:
   'ultraviolet radiation'/de OR 'sunlight'/de OR 'sun exposure'/de OR ‘occupational exposure’/de OR 'occupation'/de OR ‘employment’/de OR 'work'/de OR ‘workplace’/de OR occupational OR 'outdoor work'
4. #1 AND #2 AND #3
