# Supplementary material for: Occupational solar exposure and basal cell carcinoma. A review of the epidemiologic literature with meta-analysis focusing on particular methodological aspects
Source: Eur J Epidemiol. 2024 Jan 3;39(1):13–25. doi: 10.1007/s10654-023-01061-w (PMC10810945; doi:10.1007/s10654-023-01061-w)
Supplement: Supplementary file 2 — Supplementary Material 2 [file 10654_2023_1061_MOESM2_ESM.docx]

# Online Resource 2: PECOS-Scheme for Title/Abstract- and Full text-Screening

Screening was performed by two researchers, with involvement of a third person when necessary. Screening was based on titles/abstracts and, if relevant, full texts of references.

|  | Inclusion | Exclusion |
| --- | --- | --- |
| Population | Total population of working age and older | Study populations with other underlying illnesses (e.g. Vitiligo, Psoriasis);  Organ Transplant Patients |
| Exposure | *Title/Abstract-Screening*:  thematic reference to UV/sun exposure;  natural UV/Sun exposure;  Occupational sun/UV exposure;  Outdoor work/job;  (even if occupational UV exposure is not mentioned here, it may still play a role in the full text)  *Full Text-Screening*: **Occupational natural UV-/sun exposure**, UV-/sun exposure through **outdoor work/job** | UV exposure from artificial sources (e.g. welding arcs, lamps, lighting systems);  Cosmic radiation (Pilots and Cabin Crew) |
| Comparator | - Workers without occupational UV exposure through outdoor work;  - general population;  - indoor worker;  - other job group/s than a specific outdoor job group under investigation |  |
| Outcome | *Title/Abstract-Screening:*   - BCC, also on specific body sites or specific histology - Non-Melanoma Skin Cancer NMSC) - Keratinocyte carcinoma   *Full Text-Screening:*  BCC  BCC on specific body sites or  BCC of specific histology | - Melanoma skin cancer  - Results for squamous cell carcinoma (SCC) only  - Results for NMSC overall only  - Studies explicitly looking at BCC as a secondary tumor |
| Study Type | Cohort Study (Population cohort studies, i.e. the total population is the cohort)  Prospective Cohort Studies  Retrospective Cohort Studies  Case control Study:  - Cases with basal cell carcinoma  - Controls without basal cell carcinoma  *Full Text:*  the control group has no other skin cancer  Letters/Comments/etc. to be excluded BUT in case they point to original studies include and label as +other | Cross-sectional studies  Case-only studies (Including when different BCC cases/skin cancer cases are compared)  Animal Studies  Mortality Studies |
| Language | *Title/Abstract-Screening*: studies with English title and/or abstract  *Full Text-Screening:*  All languages |  |
