# Supplementary material for: Occupational solar exposure and basal cell carcinoma. A review of the epidemiologic literature with meta-analysis focusing on particular methodological aspects
Source: Eur J Epidemiol. 2024 Jan 3;39(1):13–25. doi: 10.1007/s10654-023-01061-w (PMC10810945; doi:10.1007/s10654-023-01061-w)
Supplement: Supplementary file 3 — Supplementary Material 3 [file 10654_2023_1061_MOESM3_ESM.docx]

# Online Resource 3: Information on meta-(regression) analyses

*Computations of risk estimates and/or 95% confidence intervals for original studies in order to use them in the analyses*

For three studies, risk estimates and/or exact 95% confidence intervals were computed based on descriptive information in the publications [27, 33, 47]. In addition, we inverted the risk estimates by Lindelöf et al. [25] for the comparison of clerical workers (i.e. largest indoor job group) with the combined reference group of farmers, foresters and gardeners. For Hannuksela-Svahn et al. [21], risk estimates with two decimal places were computed based on the published risk estimates with one decimal place, assuming the largest possible width of 95% confidence intervals.

*Complete exclusion of studies from the analyses*

Studies were excluded due to the following reasons:

- secondary analyses of data
- major restrictions of studies that limit their suitability to answer the research question whether occupational solar exposure increases BCC risk (young adult age of cases; very specific BCC)
- risk estimates and/or confidence intervals not reported and not estimable based on reported data
- massive deficits with respect to study conduct or reporting
- results for intermittent types of exposure only

*Selection of risk estimates for the analyses*

Risk estimates were selected as follows:

- in case of several analyses of the same data, risk estimates based on the most informative exposure variable (see following bullet points) or the most extensive series of cases/incidence period were used
- risk estimates for the highest reported exposure levels were used
- in case of risk estimates for different strata (e.g. men and women) or different BCC subtypes, all estimates were used
- risk estimates for occupational solar exposure due to overall outdoor work were preferred
- in terms of specific occupations, only risk estimates for agricultural jobs were considered; this group was evaluated in almost all studies on specific occupations and entails many outdoor workers; this approach ensures a certain homogeneity with regard to the index exposure among the studies on specific occupations/jobs
- when only risk estimates for different agricultural job subgroups were published, all of the reported estimates were used
- in general, the most comprehensively adjusted risk estimates were used
