# Supplementary material for: Occupational solar exposure and basal cell carcinoma. A review of the epidemiologic literature with meta-analysis focusing on particular methodological aspects
Source: Eur J Epidemiol. 2024 Jan 3;39(1):13–25. doi: 10.1007/s10654-023-01061-w (PMC10810945; doi:10.1007/s10654-023-01061-w)
Supplement: Supplementary file 4 — Supplementary Material 4 [file 10654_2023_1061_MOESM4_ESM.docx]

# Online Resource 4: PRISMA 2009 Flow Diagram

**Studies excluded, with main reasons**

Only risk estimates for intermittent exposures (n = 1)

Serious flaws (n = 4)

Certain limitations with respect to study design/aim (n = 4)

No data for risk estimates and/or confidence intervals (n = 2)

Secondary analyses of overlapping data (n = 13)

**Full-text articles excluded, with main reasons (n = 99)**
BCC not analyzed separately (n = 34)

Occupational sun exposure not analyzed separately (n = 22)

No comparison group (n = 19)

Conference papers or clinical letters (n = 15)

Wrong study design (n = 9)

Studies included in the Meta-Analysis
(n = 32)

Studies included in the Systematic Review
(n = 56)

Full-text articles assessed for eligibility
(n = 155)

Records excluded
(n = 3.603)

Records screened
(n = 3.758)

Duplicates removed
(n = 281)

Additional records identified through other sources
(n = 1)

Records identified through database searching (PubMed, Embase)
(n = 4.038)

## Included

## Eligibility

## Screening

## Identification
