# Supplementary material for: Occupational solar exposure and basal cell carcinoma. A review of the epidemiologic literature with meta-analysis focusing on particular methodological aspects
Source: Eur J Epidemiol. 2024 Jan 3;39(1):13–25. doi: 10.1007/s10654-023-01061-w (PMC10810945; doi:10.1007/s10654-023-01061-w)
Supplement: Supplementary file 5 — Supplementary Material 5 [file 10654_2023_1061_MOESM5_ESM.docx]

# Online Resource 5: Information on excluded studies/references

1. **Completely excluded studies/references**

***Secondary analyses (n=13)***

| *Secondary analyses* | *Corresponding primary analyses* |
| --- | --- |
| Bauer et al. 2021 [48], Bauer et al. 2020 [55] | Schmitt et al. 2018 [23] |
| Bogavac et al. 1998 [56] | Vlajinac et al. 2000 [54] (see below) |
| de Vries et al. 2012 [57] | Trakatelli et al. 2016 [26] |
| Green and Battistutta 1990 [58] | Green et al. 1996 [22] |
| Kaskel et al. 2015 [59] | Walther et al. 2004 [39] |
| Lock-Andersen et al. 1998 [60], Steding-Jessen et al. 2010 [51] | Kenborg et al. 2010 [34] |
| Maksimović et al. 2006 [61] | Janković et al. 2010 [62]) (see below) |
| Milán et al. 2003 [63] | Laakkonen and Pukkala et al. 2008 [19], Hannuksela-Svahn et al. 1999 [21] |
| Naldi et al. 2000 [64] | Pelucchi et al. 2007 [36] |
| Suarez et al. 2007 [65], Rosso et al. 1998 [66] | Rosso et al. 1996 [42] |

***Studies with major restrictions that limit their suitability to answer the research question whether occupational solar exposure increases BCC risk (young adult age of cases; very specific BCC) (n=4)***

**Paavilainen et al. 2005 [67]**

This study was restricted to BCC at the eyelid.

**Matas-Nadal et al. 2021 [68], Nemer et al. 2018 [69], Bakos et al. 2011 [70]**

These studies were restricted to BCC diagnoses in patients ≤40 years of age, <40 years of age and ≤45 years of age, respectively. BCC is rare in young adults. In the three studies, only few cases were included (25 patients, 50 patients and 69 patients, respectively).

***Risk estimates and/or confidence intervals not reported and not estimable based on reported data (n=2)***

**Marks et al. 1989 [71], Gon and Minelli 2011 [72]**

***Other reasons for exclusion (n=6)***

Other reasons for exclusion were mainly

- Massive deficits with respect to study conduct or reporting or
- Results for intermittent types of exposure only

Most of the studies were included in at least one previous systematic review/meta-analysis, despite the study by Vornicescu et al. 2020 [73].

**Tobia et al. 2007 [15]**

This case-control study did not meet our inclusion criteria. It compared BCC patients with patients with melanoma and SCC. Further to that, the study report lacks proper description of study conduct and data analysis.

**Lear et al. 1997 [74]**

This case-control study matched 403 cases from general dermatology outpatient clinics in the English Midlands and 403 controls with seborrhoeic warts and eczema

according to age and sex. For the variable “outdoor occupation”, no odds ratio was reported due to non-significance. An odds ratio should also not be calculated from the information in table 1 of the publication; obviously, exposure data were missing for many study subjects, with different magnitudes of deficiency for different variables and for cases and controls; this was, however, not mentioned in the publication.

**Vlajinac et al. 2000 [54]**

In this case-control study with 200 cases and 399 controls from Belgrad und Zrenjanin in Serbia, “outdoor work during summer” was identified as a risk factor for BCC (22 exposed cases; 9 exposed controls). All previous systematic reviews used the risk estimate for this variable, although the study authors discussed that it describes “intermittent” exposure in subjects that help their relatives in agriculture only in summer. In this study, another 26 cases (13%) and 69 controls (17.3%) were regular “agricultural workers”– indicating no increase in BCC risk. This, in turn, contradicts the observation of Bogavac et al. 1998 [56] of 16 farmers among 100 cases and zero farmers among 100 controls from the study centre in Belgrade. Due to the mentioned reasons, the study was excluded.

**Janković et al. 2010 [62]**

This study was obviously done along the lines of Vlajinac et al. 2000 [54]. The authors again examined the risk factor “Outdoor work during the summer-time”, which involves intermittent exposure instead of regular occupational UV exposure. Moreover, there are several conflicting information in table 2 of the publication; the odds ratios do not match the distribution of variables in cases and controls. The results, including the conflicting information in table 2, are identical to the results of a previous analysis that was published in Serbian language by Maksimović et al. 2006 [61].

**Lichte et al. 2010 [75]**

For this study, 283 male mountain guides were recruited during mountain guide reunions. Head, neck and forearms were examined for suspected skin cancer. In a nested case-control study, 20 mountain guides with histologically or clinically confirmed BCC were compared with mountain guides without BCC. Multiple regression analysis with backward elimination of non-significant variables was used to identify relevant risk factors. In this process, “guiding days” obviously prevailed over age. However, it is evident that the age of a mountain guide is highly correlated with guiding days. The odds ratio reported for guiding days is not usable as a large age difference must be assumed between cases and controls. Further to that, mountain guides are a very specific subgroup of outdoor workers whose UV exposure patterns, including their risks of sunburns, possibly differ from that of other outdoor workers. This is yet another reason why this specific study is not suitable for a meta-analysis on the risk of BCC in general outdoor workers. The study itself suggests that heavy sunburns are an important risk factor for BCC in mountain guides.

**Vornicescu et al. 2020 (73)**

This pilot study includes 52 BCC cases and 59 controls with non-malignant dermatologic conditions recruited in a hospital in Cluj-Napoca, Romania. Cases were either consecutively admitted patients with single BCC or patients who earlier had a diagnosis of BCC and had either multiple or recurrent tumors. Subjects were asked whether they had worked outdoors for more than five years. At recruitment, the mean age of cases and controls was similar (71 and 73.1 years, respectively). However, the median age of cases at their first (and possibly only) diagnosis was 66 years, while the basis for exposure assessment presumably was recruitment into the study. Thus, this study was excluded. The study was also not adjusted for age and sex and response rates were not reported.

1. **Studies that were used only for the first level of meta-analysis but were excluded at subsequent levels**

As described in the main part of the publication, studies were excluded after the first meta-analysis due to deficits with regard to data analysis. This applies to studies that were not controlled for age, sex, and study centre (if applicable) in a statistical (regression) model of the exposure-disease association. We also looked for other model-misspecifications, e.g. the inclusion of potentially highly correlated variables in the same regression model. The excluded studies and the specific reasons for their exclusion are as follows:

**Hogan et al. 1989 [47]**

For this study, cases from the cancer registry of Saskatchewan, Canada, and controls from the Provincial Medicare Plan were matched according to age, sex and residence.

A multivariable logistic regression model was built using a stepwise selection procedure of variables. “Farming as occupation” was one of the variables retained in the final model. It is unclear whether the analysis controlled for the matching factors (either by a conditional model or an unconditional model adjusted for the matching factors). Thus, the study was used only in the first meta-analysis.

**Gafà et al. 1991 [46]**

In this study, cases from Ragusa cancer registry in Sicily and controls from the same hospitals as the cases as well as from the circle of friends of the cases were matched according to age and sex.

As the crude odds ratio for the variable “Working in agriculture ≥10 years” was calculated without statistical control for the matching factors, this study was used only in the first meta-analysis.

**Walther et al. 2004 [39]**

In this study, 213 cases with a BCC diagnosis that was up to five years old were recruited in dermatology clinics in Dresden and Ulm, Germany. About 411 controls stem from the same dermatology clinics as well as from clinics of general surgery.

It is unclear whether the variables examined as risk factors for BCC in this study were censored with age at diagnosis in the case group. After univariate analyses of all possible risk factors in association with BCC, a multivariable logistic regression model was generated using backward selection. The final model included the variable “Occupational UV exposure (frequent/sometimes vs. rare/never)“, amongst others. However, due to missing data, for this model only 69% of cases and 64% of controls could be used. Furthermore, cases were on average 11 years older at diagnosis than controls at recruitment. The authors simply adjusted for age linearly, although the association between age and BCC is not linear. The final model did also not include sex. Due to the mentioned deficiencies, the study was only included in the first meta-analysis.

**Dessinioti et al. 2011 [32]**

This study includes 199 cases recruited from the oncology unit of a hospital in Athens, Greece. Controls are 200 patients with minor gastrointestinal, orthopaedic, or skin disorders, and unrelated healthy relatives.

For the potential risk factors, including “>5 years outdoor work”, the authors presented age- and sex-adjusted odds ratios, odds ratios that were additionally adjusted for constitutional factors and sun exposure patterns, and odds ratios based on a model derived with backward selection. The two latter models were clearly misspecified. For example, they both contained the variables Fitzpatrick skin type as well as skin colour at the same time. Cases were on average 14 years older than controls. Details on the statistical adjustment for age are not given. It must be assumed that age was modelled linearly which is not correct as the association between age and BCC is not linear. Due to the mentioned reasons, the study was only used in the first meta-analysis.

**Caccialanza et al. 2012 [29]**

In this study in Italy, 504 BCC patients considered for radiotherapy and 475 controls with skin diseases not related to BCC or sun exposure were matched for age and sex. Matching was not described in detail. It is unclear at which institution(s) the cases and controls were recruited. The cases have already been described earlier by Percivalle et al. 2005 [76]; afterwards, the recruitment of controls took place.

The risk modeling based on a probably used logistic regression model is particularly problematic. In addition to age and phototype (but not sex), sun exposure in childhood (yes vs. no), recreational sun exposure (yes vs. no) and occupational sun exposure for at least 6 months (yes vs. no) each entered as binary variables without any further explanation of the variables. Furthermore, total sun exposure in hours per year entered the model as a linear term. In addition to the missing description of its assessment, this variable was certainly (highly) correlated with some of the other variables in the model. Unfortunately, an odds ratio for it was not reported. Due to the mentioned reasons, the study was only used in the first meta-analysis.

**Sánchez et al. 2012 [31]**

In this study in Colombia, 203 cases from the National Referral Centre for skin diseases were compared with 203 other patients from the same dermatological centre, with matching for age. Cases and controls were not matched for sex. The multivariable conditional logistic regression model was also not adjusted for sex.

**Asgari et al. 2010 [33]**

This case-control study was nested in the population of members of the health insurance company Kaiser Permanente Northern California (KPNC) that had completed a Multiphasic Health Checkup (MHC) from 1968-1970. A total of 220 cases with BCC diagnoses from 1974-1979 and 220 controls with white skin color were matched according to age, sex, location and date of the MHC, and a few other variables.

Only crude information on the distribution of „occupational sun exposure“ in cases and controls was given. We computed a crude risk estimate that was only used in the first meta-analysis.

**Atis et al. 2015 [27]**

The 144 cases with BCC and 56 cases with other skin cancer from Dermatology and Plastic Reconstructive and Aesthetic Surgery outpatient clinics in Istanbul were at least 65 years old and matched according to age and sex with 75 healthy volunteer control subjects. Recruitment and matching were not described in detail. It is also unclear when and how exposure data were assessed.

The study authors analyzed the distributions of variables in cases and controls with simple univariate methods (e.g. Chi-Square test), amongst them the binary variable “Working outdoor” (present vs. absent). We computed a crude odds ratio and used it only in the first meta-analysis.

**Ruiz Lascano et al. 2005 [38]**

For this study, 88 consecutive BCC cases were recruited in the dermatology department of a private hospital in Córdoba, Argentina. The 88 controls without history of skin cancer stem from the medical clinic service of the same private hospital; their indications and the procedure of their recruitment were not reported. Cases and controls were matched for age and sex but details are unclear.

Continuous occupational sun exposure was assessed (details not further described) and classified as high/medium and low for data analysis. The data were analysed with unconditional logistic regression, adjusted for skin phototype. This is why we used this study only in the first meta-analysis.
