# Supplementary material for: Occupational solar exposure and basal cell carcinoma. A review of the epidemiologic literature with meta-analysis focusing on particular methodological aspects
Source: Eur J Epidemiol. 2024 Jan 3;39(1):13–25. doi: 10.1007/s10654-023-01061-w (PMC10810945; doi:10.1007/s10654-023-01061-w)
Supplement: Supplementary file 6 — Supplementary Material 6 [file 10654_2023_1061_MOESM6_ESM.docx]

# Online Resource 6: Evaluation of the literature

## *Study type*

Most of the 32 studies that are part of the meta-(regression) analyses refer to case-control studies (n = 25) (see Table 1, column 1). Five reports refer to cohort studies. Two studies conducted in Germany were classified as cancer registry-based studies irrespective of their case-control or cohort design [16, 17]. In these studies, both outcome and exposure information were retrieved from cancer registries. Job information was missing for 48-70% of registered BCC cases in the two studies.

## *Selection of subjects (Selection bias)*

We allocated a high risk of selection bias to sixteen studies (see Table 1, columns 3-6). For nine case-control studies, participation rates of cases and/or controls are unknown, and four had participation rates lower than 50%. For ten case-control studies, the type of control subjects was rated inappropriate. Most of the case-control studies were affected by both shortcomings. For the cancer registry-based studies in Germany [16, 17], a high risk of selection bias was allocated due to the high degree of missing exposure information among registered cases. There was no cohort study with a high risk of selection bias. The study by Green et al. [22] had a comparably high loss to follow up of 20%.

## *Exposure variables*

Seven studies compared the specific job group of farmers (including or excluding fishers and foresters) with all other job groups/the general population (see Table 1, column 7). The study by Lindelöf et al. [25] used farmers, foresters and gardeners as the reference group for separate comparisons with all other occupational groups, e.g. clerical workers. All other studies refer to outdoor work as the index exposure and define the comparator as no or little outdoor exposure. Fifteen studies used quantitative estimates of outdoor work as exposure variables (including on study with reference to work in agriculture) (see Table 1, column 8).

## *Outcome variables*

For seven studies, it was explicitly stated that only first incident cases were included (see Table 1 column 9). For the other studies, it cannot be ruled out that incident cases already had a previous BCC. This also holds true for studies based on cancer registries. For example, in the Finish cancer registry, a subsequent BCC at another location than the first one is registered as a separate primary BCC when the two lesions occur one year apart from each other [21].

## Data analysis

We rated nine studies to be affected by deficits regarding data analysis (see Table 1). The studies were not controlled for age, sex, and/or study centre (if applicable) and/or suffered from other misspecifications of the analytical models, e.g. the inclusion of highly correlated variables in the same regression model. More information on the affected studies is given in Online Resource 5, section B.
