# Supplementary material for: Occupational solar exposure and basal cell carcinoma. A review of the epidemiologic literature with meta-analysis focusing on particular methodological aspects
Source: Eur J Epidemiol. 2024 Jan 3;39(1):13–25. doi: 10.1007/s10654-023-01061-w (PMC10810945; doi:10.1007/s10654-023-01061-w)
Supplement: Supplementary file 7 — Supplementary Material 7 [file 10654_2023_1061_MOESM7_ESM.docx]

# Online Resource 7: Overview of case-control studies

**Table. Case-control studies (sorted in ascending order by year of publication)**

| **Study (Authors, year)** | **Study participants (source; participation rates)** Cases Controls | | **Study design characteristics** | **Exposure information** | **Analysis** | **Results** |
| --- | --- | --- | --- | --- | --- | --- |
| Schmitt et al. 2018 [23], Germany; multi-centric | Dermatologists affiliated with several regional study sites; PR 76% | Regional population registries; PR 21% | Propensity-score-matching for age and sex | Lifetime occupational standard erythema dosage, SED | LR; Adjustment for age, age^2^, sex, skin type, non-occupational UV exposure | OR = 1.84 (95% CI 1.19–2.83), ≥90th percentile (≥5,870.5 SED) vs. <44^th^ percentile (0 SED) |
| Lindelöf et al. 2017 [25]; Sweden | Cancer registry; 100% | Population registry; 100% | Only people born in Sweden; Incidence density sampling matched for age and sex | Main occupation status between 31-50 years of age (register-based) | CLR adjusted for geographical area (at index date), level of income (at index date) | OR = 0.51 (95% CI 0.47-0.55), men; 0.57 (95% CI 0.52-0.64), women; Farmers, foresters and gardeners vs. clerical workers; Computation of estimates as the inverse of the values given in Lindelöf et al. 2017 |
| Kricker et al. 2017 [24]; New South Wales, Australia | Subjects with first ever NMSC in last three years at baseline assessment of ‘45 and Up Study’; PR 36% | Subjects without ever NMSC at baseline assessment of ’45 and Up Study’; PR 31% | Frequency matching for sex and 5-year age groups (at time of baseline-questionnaire) | Years of outdoor work (jobs for ≥1 year with ≥1 hour/day outdoors) | LR with adjustment for 5-year-age groups (at time of baseline-questionnaire), sex, skin colour and age at arrival in Australia, (education) | OR = 1.18 (95% CI 0.89-1.58), 30 or more years vs. no outdoor work |
| Trakatelli et al. 2016 [26]; Europe; multi-centric | Dermatology departments; no PR reported | Dermatology patients; Companions of patients; Residents/Volunteers at homes for the elderly; Nonmedical elderly friends/relatives of research personnel; no PR reported | Frequency-matching for age and sex | Years of outdoor work | LR adjusted for age, sex, skin phototype, country, smoking, sunscreen use in own country and outdoor hobbies | OR=3.32 (95% CI 2.55-4.33), >5 years vs. never outdoor work |
| Atis et al. 2015 [27]; Istanbul, Turkey | Archives of Dermatology and Plastic Reconstructive and Aesthetic Surgery outpatient clinics; no PR reported (if study solely based on clinic archives, then PR 100%) | Healthy volunteers not further specified; no PR reported | Subjects at age >65 only; Matching for age and sex | Outdoor work (no information on exact question regarding outdoor work; unclear if information stems from clinic archives or was additionally gathered from study participants) | - | OR = 1.20 (95% exact CI 0.65-2.23) for outdoor work yes vs. no; Computation of estimates based on information reported by Atis et al. 2015 |
| Surdu et al. 2013 [28]; Europe; multi-centric | Primary/private care or hospitals; PR 81.6% | General surgery in-patients and orthopaedic or trauma patients; PR 90%; | Frequency matching for county of residence, sex, and 5-year age range | Cumulative exposure to natural UV at work based on occupational history (job titles held ≥1 year over lifetime) | LR adjusted for sex, age, residence, complexion of skin, family history of cancer, and lifetime average arsenic concentration in drinking water | OR = 0.62 (95% CI 0.30-1.28), third tertile of cumulative exposure to natural UV (> 5075 hours) vs. never exposed to UV at work |
| Caccialanza et al. 2012 [29]; Milan, Italy | Patients considered for radiotherapy; no PR reported | Subjects with skin diseases not related to BCC or sun exposure; no PR reported | Matching for sex and age at diagnosis (cases)/observation (controls) | Professional sun exposure (for a minimum of 6 months) | LR; Adjusted for age, sun exposure in childhood, recreational sun exposure, Fitzpatrick photo-type, sun exposure hours/year | OR = 2.97 (95% CI 1.93-4.59), professional sun exposure yes vs. no; |
| Iannacone et al. 2012 [30]; Tampa, Florida | University of South Florida Dermatology clinic (PR 80%) and additional cases from skin cancer screening | Skin cancer screening; PR 49.7% (according to Rollison et al. 2012 [77]: (95+281)/756) | White participants only (incl. Hispanics) | Years of job in sunlight with duration of at least three months | LR using subjects with complete data (91.6% of cases); after backward elimination adjusted for age, gender, ethnicity, education (≤12 vs. >12 years?), eye and hair colour, cutaneous sensitivity, tanning ability, history of smoking | OR = 2.12 (95% CI 1.05-4.27); Job in sunlight for ≥3 months for >10 years vs. never job in sunlight for ≥3 months |
| Sánchez et al. 2012 [31]; Bogota, Colombia | National Referral Centre for skin diseases; no PR reported | Patients from the same dermatology centre; no PR reported | Matching for age | Occupational activity outdoors at age >30 | CLR adjusted for rural residence at age >30, failure to use hat at age <15, lifetime practice of outdoor sports, family history of skin cancer, history of ≥10 episodes of sunburn, history of actinic keratosis, actinic conjunctivitis, phototype I-III; | OR = 1.67 (95% CI 0.82-3.44), occupational activity outdoors at age >30 yes vs. no |
| Dessinioti et al. 2011 [32]; Athens, Greece | Oncology unit of a hospital that serves as referral centre for skin cancer; no PR reported | Unrelated healthy relatives; Patients with minor gastrointestinal, orthopaedic, or skin disorders; no PR reported | - | Years of outdoor work | LR; adjusted for sex, age, skin colour, hair colour, Fitzpatrick skin type, solar lentigines, actinic keratosis, common nevi, beach summer holidays, history of sunburns, UV protection (sunscreens, clothing) | OR = 2.7 (95% CI 1.4-5.3), >5 years vs. 0-5 years of outdoor work |
| Asgari et al. 2010 [33]; California, USA | BCC cases among members of Kaiser Permanente Northern California that had completed a Multiphasic Health Checkup (MHC) earlier; PR 100% | Non-cases among members of Kaiser Permanente Northern California that had completed a Multiphasic Health Checkup (MHC); PR 100% | Only subjects with white skin colour; Matching for age, sex, location and date of the MHC, etc. | Occupational sun exposure (assigned based on occupations), | - | OR = 1.17 (exact 95% CI 0.32-4.37), occupational sun exposure high vs. low; Computation of estimates based on information reported by Asgari et al. 2010 |
| Kenborg et al. 2010 [34]; Denmark | Cancer registry; PR 100% | Central Person Registry; PR 100% | Men only; Incidence density sampling matched for age and year of birth | Years of outdoor work (based on information on industry employed from Supplementary Pension Fund) | CLR adjusted for first known year of employment, place of birth, skin colour, and social class; lag-time for exposure 10 years | OR for BCC at head = 0.86 (95% CI 0.78, 0.95); OR for BCC at body = 0.63 (95% CI 0.52, 0.76); OR for BCC at upper extremities = 0.81 (95% CI 0.56, 1.19); OR for BCC at lower extremities = 0.45 (95% CI 0.25, 0.83); men; >10 years vs. <1 year of outdoor work |
| Marehbian et al. 2007 [35]; New Hamspire, USA | Skin cancer registry; PR 74% | Driver’s licence register and Medicare/Medicaid-files; PR 68% | Frequency-matching for 10-year-/5-year- age groups and sex | Jobs held since age 15 | LR stratified by sex; Adjusted for age (<50, 50-59, 60-69, ≥70), education, skin reaction to sun, painful sunburns, recreational sun exposure | OR = 2.0 (95% CI 0.9-4.6), ever vs. never farm operator/manager, men; OR = 1.0 (95% CI 0.7-1.5), ever vs. never other agricultural/related occupation, men |
| Pelucchi et al. 2007 [36]; Italy; multi-centric | Hospital-based dermatological or cancer centres; PR >97% | Hospitals patients with acute, non-dermatologic conditions; PR >97% | Matching for age and sex | Lifetime hours of occupational sun exposure, weighted by body area exposed (based on work history including jobs ≥6 months) | LR; adjusted for age (5-year categories), sex, study centre, education, pigmentary traits | OR = 1.35 (95% CI 0.85-2.14), nodular BCC; OR = 0.50 (95% CI 0.25-1.00), superficial BCC; >median no. of lifetime hours vs. no occupational exposure |
| Zanetti et al. 2006 [37]; Europe and Argentina; multi-centric | Clinics/hospitals/outpatient departments; PR 80.6% | Hospital patients without dermatological diseases and orthopaedic injuries; PR 92.8% | Male Caucasians only; Frequency-Matching for age | Lifetime sun irradiation-weighted hours of outdoor work (based on work history including outdoor jobs of ≥6 months duration) | LR; Adjustment for age, country/region, host factors | OR = 1.2 (95% CI 0.70-2.13), 3878+ sun irradiation-weighted hours of outdoor work vs. no outdoor work, men |
| Ruiz Lascano et al. 2005 [38]; Córdoba, Argentina | Dermatology department of a private hospital in Córdoba; PR 95% | Controls without history of skin cancer from the medical clinic service of the same hospital; PR 99% | Matching for age and sex | Occupational sun exposure high, medium and low | LR adjusted for skin phototype | OR = 2.6 (95% CI 0.60-10), Occupational sun exposure high/medium vs. low |
| Walther et al. 2004 [39]; Dresden/Ulm, Germany; multi-centric | Dermatological clinics; no PR reported | Allergology, phlebology and proctology patients from the same clinics and patients from clinics of general surgery; no PR reported | - | Frequency of occupational UV exposure (ordinal) | LR with backward selection, restricted to 69% of cases/64% of controls due to missing data; Finally adjusted for age (at diagnosis? at study participation?), study centre, non-BCC skin cancers, non-skin cancers, phenotype, precursor lesions, benign skin lesions, BCC in family, sunburns 20 years ago; | OR = 2.4 (95% CI 1.3-4.7), frequent/occasional vs. rare/never occupational UV exposure |
| Corona et al. 2001 [40]; Rome, Italy | Referral hospital for skin diseases; no PR reported | Hospital patients with minor dermatological conditions; no PR reported | - | Years of outdoor work | LR; Adjusted for age (continuous), sex, pigmentary traits, family history of skin cancer | OR = 1.7 (95% CI 0.7-4.1), >8 years vs. ≤8 years outdoor work |
| Rosso et al. 1999 [41]; Sion, Switzerland | Sion Cancer registry; PR 73% | Contributors/Supporters of the Swiss League for the Fight Against Cancer; Volunteer associations of blood donors; PR 81% | Matching for age and sex | Lifetime hours of outdoor work | CLR | OR = 0.90 (95% CI 0.51-1.59), 77 200+ lifetime hours vs. never outdoor work |
| Rosso et al. 1996 [42]; Italy, Spain, France; multi-centric | Cancer registries; Hospital patients; PR 88% | Electoral rolls/population registries; Hospital patients without skin diseases or cancer; PR 73.6% |  | Lifetime hours of outdoor work | LR; adjusted for age (continuous), sex, centre, pigmentary traits, skin reaction to sun, sunburns, holidays at beach; water sports | OR = 1.00 (95% CI 0.78-1.30),  54 720+ lifetime hours of outdoor work vs. <7200 hours |
| Gallagher et al. 1995 [43]; Alberta, Canada | Cancer registry; 72% | Subjects sampled from general population using Healthcare Subscriber lists; PR 71% | Men only; Matching by 5-year age groups | Mean lifetime occupational sun exposure hours per year weighted by clothes worn | CLR with age stratified in 1-year categories; Adjustment for skin colour, hair colour, mother's ethnic origin | OR = 1.4 (95% CI 0.8-2.4), men, ≥105 vs. <15 mean lifetime hours/year of occupational sun exposure |
| Kricker et al. 1995 [45]; Geraldton, Australia | Geraldton skin cancer survey, Nov 1987 (new cases or diagnosed in preceding year); interview in year 1988; PR 89% | Participants in the same survey without BCC; PR 89% | Frequency-Matching for sex and age (female; male 40-54 years, male 55-64 years) | Lifetime hours of sun exposure on work days from 9 to 5 in a typical week | LR, restricted to native born Australians with Northern European ancestry; Adjustment for 5-yearage intervals, sex, cutaneous sun sensitivity | OR = 0.86 (95% CI 0.50-1.51), 4^th^ quartile (≥49.4 hours) vs. 1^st^ quartile (≤14.7 hours) of sun exposure on work days in a typical week |
| Maia et al. 1995 [44]; Sao Paulo, Brazil | Dermatology clinic; no PR reported | Patients from the same dermatology clinic; no PR reported | Matching for age and sex | Previous or present agricultural activity | CLR; Final adjustment after backward variable selection for actinic changes of skin and skin type | OR = 4.9 (95% CI 3.2-7.6), Ever vs. never agricultural activity |
| Gafà et al. 1991 [46]; Ragusa, Sicily, Italy | Regional cancer registry (diagnoses in 1987 and 1988); PR 94% | Friends and relatives of cases; Patients without cancer from the same hospitals/outpatient departments as cases; no PR reported | Matching for age (for cases: age at diagnosis or exposure assessment?) and sex (two controls per case) | Years of work in agriculture  Exposure assessment in 1990; unclear if censoring with BCC diagnosis | Odds Ratios using Woolf approximation for CI | OR = 1.6 (95% CI 1.02-2.5); ≥10 years vs. <10 years or never work in agriculture |
| Hogan et al. 1989 [47]; Sasketchewan, Canada | Provincial Cancer registry; PR 55.5% | Provincial Medicare Plan; PR 43.7% | Matching for birth year, sex, rural municipality of residence  Authors stated 1:2-Matching but 538 cases/0.555 is more than half of 738/0.437 | Farming as occupation  Exposure assessment seems to focus on situation at interrogation; unclear when interview was held | LR using stepwise regression; finally adjusted for host factors, Irish/Scottish/Welsh origin of mother, family history of skin cancer, sunburn history, working outdoors >3 hours/day in winter | OR = 1.29 (95% CI 1.13-1.47), occupation as farmer yes vs. no; exact 95% CI computed from information on logOR and logSE reported by Hogan et al. 1989 |

CLR = conditional logistic regression; LR = logistic regression; NMSC = non-melanoma skin cancer
