# Supplementary material for: Occupational solar exposure and basal cell carcinoma. A review of the epidemiologic literature with meta-analysis focusing on particular methodological aspects
Source: Eur J Epidemiol. 2024 Jan 3;39(1):13–25. doi: 10.1007/s10654-023-01061-w (PMC10810945; doi:10.1007/s10654-023-01061-w)
Supplement: Supplementary file 8 — Supplementary Material 8 [file 10654_2023_1061_MOESM8_ESM.docx]

# Online Resource 8: Overview of other studies

Table. Characteristics of other studies (non-case-control studies)

| **Study (Authors, year)** | **Type of study;  Setting** | **Outcome  definition** |  | **Exposure information** | **Analysis** | **Risk estimates** |
| --- | --- | --- | --- | --- | --- | --- |
| Radespiel-Tröger et al. 2009 [16] | Cancer registry-based study; Eleven Bavarian districts, Germany | First incident BCC, 2001-2005, ascertained from cancer registry | | Notifications to cancer registry on longest job or, if not available, on current job by medical professions/authorities based on declarations of patients  Exposure information missing for 70% of registered female and 55.5% of registered male cases | Comparison of age-standardized incidence rates for outdoor vs. indoor workers, using population counts of the years of diagnoses weighted by the proportion of outdoor and indoor work (derived from official statistics for Bavaria) and the availability of job notifications in the study as denominators | IRR = 2.9 (95% CI 2.2-3.9), men, outdoor vs. indoor work  IRR = 2.7 (95% CI 1.8-4.1), women, outdoor and mixed outdoor/indoor vs. indoor work |
| Seidler et al. 2006 [17] | Cancer registry-based study; Rhineland-Palatinate, Germany | Incident BCC, 1998-2003, ascertained from cancer registry | | Notifications to cancer registry on longest and last job by medical professions/authorities based on declarations of patients  Exposure information missing for 49.5% of males and 47.7% of females with BCC as well as 56.8% of males and 48.4% of females with other cancer types | Age-adjusted odds ratios comparing BCC cases with all other cancer cases with respect to several outdoor occupations | OR for ever being a farmer or farm worker vs. white-collar or production workers (excluding outdoor workers) 1.1 (95% CI 0.9-1.3), men; 1.5 (95% CI 1.2-1.8), women |
| Laakkonen and Pukkala 2008 [19] | Cohort study; Finnish population | Incident BCC, 1995-2005, ascertained from cancer registry | | Finnish Farm Registry: Male farm owners on 31 December 1978; Farmers’ Social Insurance Institution: female farmers from these farms | Age- and sex-standardized incidence rate ratios comparing farmers with general population, using person-time derived from population registry | SIR = 0.69 (95% CI 0.63-0.74) for farmers still farming in 1990 or 1994  SIR = 0.80 (95% CI 0.77-0.82) for farmers that quit farming in 1990 or 1994 |
| Hannuksela-Svahn et al. 1999 [21] | Cohort study; Finnish population born from 1906-1945 | Incident BCC, 1971-1995, ascertained from cancer registry | | Occupation at the time of the Finnish Census at last day in 1970 | Age-standardized incidence rate ratios comparing occupational groups with general population, using person-time derived from population registry | SIR for Farming, fishing, forestry 0.84 (95% CI 0.75-0.95), men; 0.90 (95% CI 0.85-0.95), women; estimators re-computed based on the values with one decimal place published by Hannuksela-Svahn et al. 1999, assuming the largest possible width of confidence intervals |
| Cai et al. 2010 [18] | Prospective cohort study in various Japanese public health center areas, with participants 40-69 years of age and without cancer history at baseline | First NMSC (including BCC), 1990-2013, ascertained from hospitals and cancer registries, with supplementary information from death certificates; LFUP 0.11% | | Self-report of pre-defined occupational types by study participants in baseline questionnaires | Hazard ratios adjusted for age, public health center, alcohol consumption, coffee consumption, smoking status, physical activity, body mass index, family history of cancer, and green tea, Chinese tea, black tea consumption | Outdoor work (agriculture, fishing, forestry) vs. indoor work (all other jobs)  Men: 1.13 (95% CI 0.58-2.21); Women: 0.74 (95% CI 0.42-1.28) |
| Green et al. 1996 [22] | Members of Nambour electoral roll, Australia, taking part in skin cancer survey in 1986 and further examinations/inquiries until 1992 | Incident BCC, Dec 1985-Mar 1992, ascertained by skin examinations**^a^** (histologically verified) and inquiry**^b^** (verification with medical records); LFUP 20% at latest contact in 1992 | | Questionnaire at survey in 1986; inquiry of present and past occupations (whether mainly outdoors, indoors, or a mixture of indoors and outdoors) | Incidence rate ratios based on first BCC in study period adjusted for age, sex, skin colour | IRR = 1.25 (95% CI 0.88-1.78), Mainly outdoor vs. mainly indoor work at present and in the past |
| Neale et al. 2007 [20] | Members of Nambour electoral roll, Australia, taking part in skin cancer survey in 1986 (see Green et al. 1996 [22]; 1990 [58]) and intervention study from 1992-96 | Incident BCC at head or trunk, 1992-2004, ascertained by skin examination**^c^** (histologically verified) and self-reports verified by medical records as well as notifications from pathology laboratories | | Lifetime occupational sun exposure, assessed in 1992 at start of intervention study: mainly outdoors, outdoors and indoors, or mainly indoors | Odds ratios adjusted for age in 1992 and sex (adjustments for trial interventions, beta-carotene and sunscreen use, did not alter any risk estimates) | Lifetime occupations mainly outdoors vs. mainly indoors  OR=0.86 (95% CI 0.53-1.40) for BCC at head/neck  OR=1.12 (95% CI 0.60-2.11) for BCC at trunk |

LFUP = Loss to follow-up

**^a^** 1986: whole body for 10%, face, neck, upper limbs for 90%; Feb/Mar 1992: whole-body

**^b^** Inquiry covering time periods Dec 1985-Nov 1987, Dec 1987-Aug 1990, Jan 1991-Jan 1992

**^c^** Skin examinations in the years 1994 and 1996; in 2000 for a subset of subjects
