# Supplementary material for: Occupational solar exposure and basal cell carcinoma. A review of the epidemiologic literature with meta-analysis focusing on particular methodological aspects
Source: Eur J Epidemiol. 2024 Jan 3;39(1):13–25. doi: 10.1007/s10654-023-01061-w (PMC10810945; doi:10.1007/s10654-023-01061-w)
Supplement: Supplementary file 9 — Supplementary Material 9 [file 10654_2023_1061_MOESM9_ESM.docx]

Online Resource 9

**Table.** Pooled effect estimates by random effects meta analysis among case-control studies without deficits regarding data analysis

|  | **Adequate control participants** | **Inadequate control participants** |  |
| --- | --- | --- | --- |
|  |  |  |  |
| **Response rate ≥50%** | n = 9 studies  0.81 (95% CI 0.66-0.98) | n = 1 study**^a^**  - | n = 10 studies  0.81 (95% CI 0.67-0.97) |
|  |  |  |  |
| **Response rate <50%/missing** | n = 2 studies**^b^**  - | n = 4 studies**^c^**  3.10 (95% CI 2.06-4.68) | n = 6 studies  2.28 (95% CI 1.46-3.56) |
|  |  |  |  |
|  | n = 11 studies  0.87 (95% CI 0.71-1.07) | n = 5 studies  2.30 (95% CI 1.27-4.16) | n = 16 studies  1.08 (95% CI 0.83-1.41) |

**^a^** The case-control study by Rosso et al. 1999 [41] recruited control participants from the sources of contributors/supporters of the Swiss League for the Fight Against Cancer and volunteer associations of blood donors; the publication entails no information if these sources represent the general population properly or not; thus, the controls were not classified as adequate. The risk estimate of this study was 0.90 (95% CI 0.51-1.59).

**^b^** As only two studies are available, no pooled risk estimates is reported. The studies by Schmitt et al. 2018 [23] and Kricker et al. 2017 [24] have study-specific risk estimates of 1.84 (95% CI 1.19-2.83) and 1.18 (95% CI 0.89-1.58), respectively. The study by Schmitt et al. 2018 recruited cases via dermatologists and controls from population registries, with participation rates of 78% and 21%, respectively. The study by Kricker et al. 2017 includes cases and controls that indicated incident BCC status at the baseline assessment of a prospective cohort study. A total of 36% of those with BCC and 31% of those without BCC took part. This study might be less affected from selection bias based on its particular study design. However, the low participation rates and the lack of clarity if participants differ from non-participants led to the classification of „high risk of selection bias“.

**^c^** All four studies with inadequate controls and inadequate response rates included dermatologic control subjects [26, 30, 40, 44]. The study by Trakatelli et al. 2016 [26] further included controls that were companions of patients, residents/volunteers at homes for the elderly, or nonmedical elderly friends/relatives of research personnel.
