# Supplementary material for: Occupational solar exposure and basal cell carcinoma. A review of the epidemiologic literature with meta-analysis focusing on particular methodological aspects
Source: Eur J Epidemiol. 2024 Jan 3;39(1):13–25. doi: 10.1007/s10654-023-01061-w (PMC10810945; doi:10.1007/s10654-023-01061-w)
Supplement: Supplementary file 10 — Supplementary Material 10 [file 10654_2023_1061_MOESM10_ESM.docx]

Online Resource 10

**Table.** The influence of specific issues on risk estimates of studies on the association between occupational solar exposure and BCC. Results based on separate univariable meta-regression analyses, restricted to studies without deficits regarding data analysis

| **Comparison** | **All studies** | | | **Case-control studies** | | |
| --- | --- | --- | --- | --- | --- | --- |
|  | **Risk estimate** | **Lower 95%-CI** | **Upper 95%-CI** | **Risk estimate** | **Lower 95%-CI** | **Upper 95%-CI** |
| Studies with high vs. low risk of selection bias**^a^** | 2.32 | 1.74 | 3.08 | 2.48 | 1.62 | 3.79 |
| Studies with quantitative vs. other exposure variables**^a^** | 0.94**^b^** | 0.64 | 1.38 | 0.92**^b^** | 0.49 | 1.75 |
| Studies that compare specific single outdoor occupations with all other occupations/the general population vs. all other studies**^a^** | 1.00 | 0.65 | 1.54 | - **^c^** |  |  |
| Studies with BCC cases that were explicitly first ever BCC cases versus other studies**^a^** | 0.98 | 0.66 | 1.45 | 0.72 | 0.42 | 1.23 |

**^a^** The categorization of the studies is shown in Table 1. **^b^** The regression coefficient and 95% confidence interval is the same for studies both with cumulative exposure variables and without exposure reference groups containing “all other occupations”/the general population as compared to all other studies.
**^c^** Not computed as only two studies were in the subgroup of studies that compare specific single outdoor occupations with all other occupations/the general population
