# Supplementary material for: Occupational solar exposure and basal cell carcinoma. A review of the epidemiologic literature with meta-analysis focusing on particular methodological aspects
Source: Eur J Epidemiol. 2024 Jan 3;39(1):13–25. doi: 10.1007/s10654-023-01061-w (PMC10810945; doi:10.1007/s10654-023-01061-w)
Supplement: Supplementary file 11 — Supplementary Material 11 [file 10654_2023_1061_MOESM11_ESM.docx]

Online Resource 11

**Figure.** Meta-analyses of studies on the association between occupational solar exposure and the risk of basal cell carcinoma. First analysis based on studies with low RoB acc. to WHO/ILO 2021 [4] (Fig. 5, page 56) and low RoSB according to our criteria. Second analysis based on studies with high RoB in at least one dimension acc. to WHO/ILO 2021 [4] (Fig. 5, page 56)

***Comment:*** Studies with low RoB according to WHO/ILO working group [4] (Fig. 5, page 56) and low RoSB according to our criteria have a pooled risk estimate of ***0.98 (95% CI 0.76-1.26)***. Studies with a high RoB in any dimension according to WHO/ILO working group [4] (Fig. 5, page 56) have a pooled risk estimate of ***1.67 (95% CI 1.12-2.49)***.

Two studies with low RoB according to WHO/ILO working group [4] (Fig. 5, page 56) had a high RoSB according to our criteria [23, 30]. If these studies were allocated to the stratum of studies with high RoB in at least one dimension, the pooled risk estimate of this stratum would be ***1.73 (95% CI 1.25-2.40)***.
